# The complete mitochondrial genome of *Plumarella spinosa* (Octocorallia: Calcaxonia: Primnoidae) from South Korea

**DOI:** 10.1080/23802359.2020.1715275

**Published:** 2020-01-20

**Authors:** Eun Hwa Choi, ChoRong Shin, Su Youn Baek, Jihye Hwang, Jumin Jun, Keum Hee Jang, Shi Hyun Ryu, Ui Wook Hwang

**Affiliations:** a Department of Biology Education, Teachers College & Institute for Phylogenomics and Evolution, Kyungpook National University, Daegu, Republic of Korea;; b School of Life Science, Kyungpook National University, Daegu, Republic of Korea;; c Institute for Korean Herb-Bio convergence promotion, Kyungpook National University, Daegu, Republic of Korea;; d Animal Resources Division, National Institute of Biological Resources, Incheon, Republic of Korea;; e Research Center for Endangered Species, National Institute of Ecology, Seocheon-gun, Republic of Korea;; f Freshwater Biodiversity Research Division, Nakdonggang National Institute of Biological Resources, Sangju, Republic of Korea

**Keywords:** *Plumarella spinosa*, Primnoidae, mitochondrial genome, phylogenetic analysis

## Abstract

*Plumarella spinosa* (Anthozoa: Octocorallia: Primnoidae) is an endangered marine soft coral species discovered on a 50–100 m deep reefs in South Korea. We analyzed the mitochondrial genome sequence of this species. The genome size was 19,037 bp in length consisting of 14 protein coding genes (PCGs), two rRNA genes and a tRNA gene. Our phylogenetic analysis for this species with 33 Octocorallia species reconstructed based on the nucleotide sequences of 14 PCGs showed that *P. spinosa* was placed as a sister to *Narella hawainensis* and Primnoidae formed a monophyletic group.

*Plumarella spinosa* (Anthozoa: Octocorallia: Primnoidae) is one of three *Plumarella* species that has reported to be found in South Korea (The National Institute of Biological Resources 2019). There are about 40 species in this genus worldwide. Since this species is symbiotic with many other marine species, it is very important in the functional aspects of the marine ecosystem. Moreover, *P. spinosa* is enrolled as an endangered species in South Korea (The National Institute of Biological Resources 2019). In this regard, its detailed genetic information is necessary to understand the distribution, ecological stability and population structure.

We analyzed the complete mitochondrial genome of this species (GenBank accession no. MG573069) which collected from Sasu-do, Jeju-si, Jeju-do, South Korea (33°55′05″N, 126°38′21″E). The specimen was deposited in the National Institute of Biological Resources, Incheon, Republic of Korea under the voucher number TNMIIV0000000007. DNA was extracted according to the manufacturer’s instruction using QIAamp Tissue Kit (Qiagen, Valencia, CA), and the mitochondrial genome was characterized by primer-walking using long template PCR products (Roche, co. Germany). Sequences were aligned, trimmed and assembled using the Clustal W program implemented in BioEdit 7.0.9 (Hall 1999). A maximum likelihood (ML) analysis was constructed with IQ-Tree (Nguyen et al. 2015).

The genome size was 19,037 bp in length being composed of 14 PCGs, two rRNA genes (*16S rRNA*, *12S rRNA*) and a single tRNA gene (*trnM*). Its G + C content was 37.92%, which is a common mitochondrial feature in Octocorallia (Park et al. 2012). *COX3*, *ATP6*, *ATP8* and *COX2* genes were located on light strand, whereas *COX1*, *ND1*, *CYTB*, *ND6*, *ND3*, *ND4L*, *mutS*, *ND2*, *ND5* and *ND4* on the heavy strand, which is the mitogenome arrangement pattern normally found in Alcyonnidae, Briareidae, Geogoniidae, Nephtheidae and Plesauridae in Octocorallia Only difference was found in that the *trnM* gene was located in the light strand, which was identified in *Narella hawaiinensis* in Primnoidae. It is also characteristic of Octocorallia that this species has only a single tRNA site and inter-genic sequences (IGSs) that were believed to be traces of the intron regions. All PCGs were initiated by ATG and terminated with TAA or TAG with the exception of *COX1* that were ended with incomplete stop codon.

The phylogenetic tree was reconstructed with 14 PCGs to identify the evolutionary placement among 33 species in Octocorallia. *Briaerum asbestinum* (NC_008073) was used as outgroup. The phylogenetic tree supported two major clades within the Octocorallia. One clade (clade A) included the Alcyoniina and Holaxonia, while the other divides into two branches, one (clade B-1) composed of Scleraxonia and Alcyoniina, and the second (clade B-2) with Calcaxonia (Figure 1). Calcaxonia formed a polyphyletic group. *P. spinosa* was placed in the clade B-2 and was a sisiter to *Narella hawainensis.* The phylogenetic tree also supported the family Primnoidae to form a monophyly.

**Figure 1.**
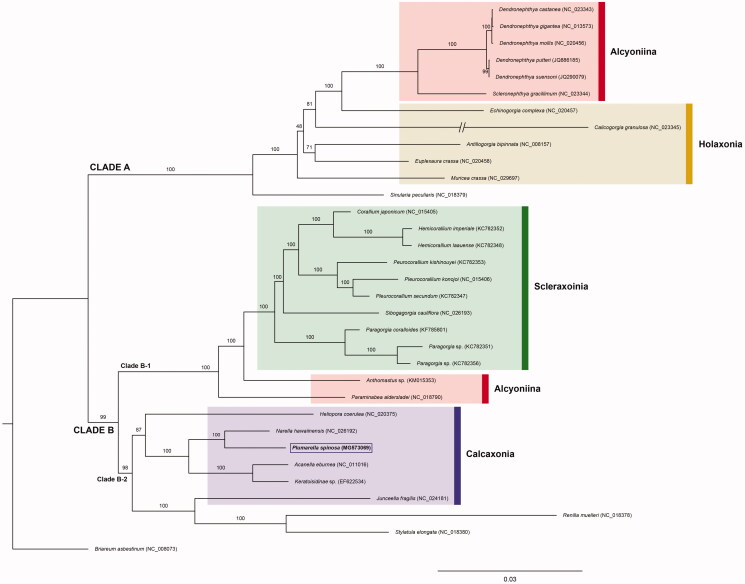
Maximum likelihood phylogenetic trees of 33 octocoralians (GenBank numbers in tip labels) based on the amino acid sequences of concatenated 14 mitochondrial protein coding genes (PCGs). Tree was reconstructed with *P. spinosa* (this study). ML tree was generated with IQTree. *100/1.00(BP/BPP).
